# 2-Chloro-7,8-dimethyl­quinoline-3-carbaldehyde

**DOI:** 10.1107/S1600536809040860

**Published:** 2009-10-10

**Authors:** F. Nawaz Khan, R. Subashini, Atul Kumar Kushwaha, Venkatesha R. Hathwar, Seik Weng Ng

**Affiliations:** a Chemistry Division, School of Science and Humanities, VIT University, Vellore 632 014, Tamil Nadu, India; b Solid State and Structural Chemistry Unit, Indian Institute of Science, Bangalore 560 012, Karnataka, India; c Department of Chemistry, University of Malaya, 50603 Kuala Lumpur, Malaysia

## Abstract

All the non-H atoms of the title compound, C_12_H_10_ClNO, lie on a crystallographic mirror plane orientated perpendicular to the crystallographic *b* axis.

## Related literature

For a review of the synthesis of quinolines by the Vilsmeier–Haack reaction, see: Meth-Cohn (1993 ▶).
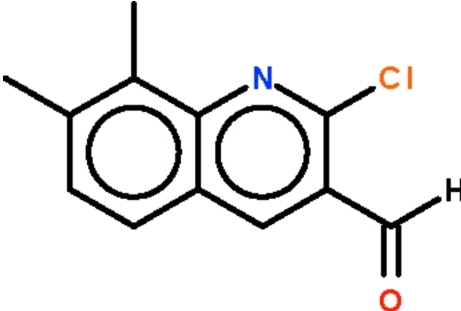


## Experimental

### 

#### Crystal data

C_12_H_10_ClNO
*M* *_r_* = 219.66
Orthorhombic, 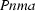

*a* = 20.4542 (13) Å
*b* = 6.7393 (4) Å
*c* = 7.5675 (4) Å
*V* = 1043.16 (11) Å^3^
*Z* = 4
Mo *K*α radiation
μ = 0.34 mm^−1^
*T* = 290 K
0.28 × 0.21 × 0.17 mm

#### Data collection

Bruker SMART area-detector diffractometer
Absorption correction: multi-scan (*SADABS*; Sheldrick, 1996 ▶) *T*_min_ = 0.912, *T*_max_ = 0.945
7145 measured reflections
1299 independent reflections
1078 reflections with *I* > 2σ(*I*)
*R*_int_ = 0.022

#### Refinement

*R*[*F* ^2^ > 2σ(*F* ^2^)] = 0.034
*wR*(*F* ^2^) = 0.115
*S* = 1.07
1299 reflections
94 parameters
H-atom parameters constrained
Δρ_max_ = 0.29 e Å^−3^
Δρ_min_ = −0.30 e Å^−3^

### 

Data collection: *SMART* (Bruker, 2004 ▶); cell refinement: *SAINT* (Bruker, 2004 ▶); data reduction: *SAINT*; program(s) used to solve structure: *SHELXS97* (Sheldrick, 2008 ▶); program(s) used to refine structure: *SHELXL97* (Sheldrick, 2008 ▶); molecular graphics: *X-SEED* (Barbour, 2001 ▶); software used to prepare material for publication: *publCIF* (Westrip, 2009 ▶).

## Supplementary Material

Crystal structure: contains datablocks global, I. DOI: 10.1107/S1600536809040860/bt5086sup1.cif

Structure factors: contains datablocks I. DOI: 10.1107/S1600536809040860/bt5086Isup2.hkl

Additional supplementary materials:  crystallographic information; 3D view; checkCIF report

## Figures and Tables

**Fig. 1.**
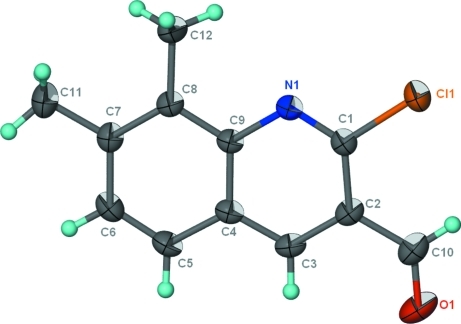
Anisotropic displacement ellipsoid plot (Barbour, 2001) of C12H10ClNO at the 50% probability level; hydrogen atoms are drawn as spheres of arbitrary radius.

## supplementary crystallographic information

### Experimental

A Vilsmeier-Haack adduct prepared from phosphorus oxytrichloride (6.5 ml, 70 mmol) and *N*,*N*-dimethylformamide (2.3 ml, 30 mmol) at 273 K was added to *N*-(2,3-dimethylphenyl)acetamide (1.63 g, 10 mmol). The mixture was heated at 353 K for 15 h. The mixture was poured onto ice; the white product was collected and dried. The compound was purified by recrystallization from a petroleum ether/ethyl acetate mixture.

### Refinement

H-atoms were placed in calculated positions (C–H 0.93–0.96 Å) and were included in the refinement in the riding model approximation, with *U*(H) set to 1.2–1.5*U*(C).

### Crystal data

**Table:**

C_12_H_10_ClNO	*F*(000) = 456
*M**_r_* = 219.66	*D*_x_ = 1.399 Mg m^−^^3^
Orthorhombic, *P**n**m**a*	Mo *K*α radiation, λ = 0.71073 Å
Hall symbol: -P 2ac 2n	Cell parameters from 753 reflections
*a* = 20.4542 (13) Å	θ = 2.0–24.4°
*b* = 6.7393 (4) Å	µ = 0.34 mm^−^^1^
*c* = 7.5675 (4) Å	*T* = 290 K
*V* = 1043.16 (11) Å^3^	Block, colorless
*Z* = 4	0.28 × 0.21 × 0.17 mm

### Data collection

**Table:**

Bruker SMART area-detector diffractometer	1299 independent reflections
Radiation source: fine-focus sealed tube	1078 reflections with *I* > 2σ(*I*)
graphite	*R*_int_ = 0.022
φ and ω scans	θ_max_ = 27.5°, θ_min_ = 2.0°
Absorption correction: multi-scan (*SADABS*; Sheldrick, 1996)	*h* = −25→26
*T*_min_ = 0.912, *T*_max_ = 0.945	*k* = −8→4
7145 measured reflections	*l* = −9→9

### Refinement

**Table:**

Refinement on *F*^2^	Secondary atom site location: difference Fourier map
Least-squares matrix: full	Hydrogen site location: inferred from neighbouring sites
*R*[*F*^2^ > 2σ(*F*^2^)] = 0.034	H-atom parameters constrained
*wR*(*F*^2^) = 0.115	*w* = 1/[σ^2^(*F*_o_^2^) + (0.0706*P*)^2^ + 0.1712*P*] where *P* = (*F*_o_^2^ + 2*F*_c_^2^)/3
*S* = 1.07	(Δ/σ)_max_ = 0.001
1299 reflections	Δρ_max_ = 0.29 e Å^−^^3^
94 parameters	Δρ_min_ = −0.30 e Å^−^^3^
0 restraints	Extinction correction: *SHELXL97* (Sheldrick, 2008), Fc^*^=kFc[1+0.001xFc^2^λ^3^/sin(2θ)]^-1/4^
Primary atom site location: structure-invariant direct methods	Extinction coefficient: 0.010 (2)

### Fractional atomic coordinates and isotropic or equivalent isotropic displacement parameters (Å^2^)

**Table:**

	*x*	*y*	*z*	*U*_iso_*/*U*_eq_	Occ. (<1)
Cl1	0.59400 (3)	0.2500	0.07517 (7)	0.0568 (2)	
N1	0.48555 (8)	0.2500	0.24995 (19)	0.0363 (4)	
O1	0.68317 (9)	0.2500	0.5872 (3)	0.0672 (5)	
C1	0.54815 (9)	0.2500	0.2711 (2)	0.0369 (4)	
C2	0.58228 (10)	0.2500	0.4343 (3)	0.0385 (4)	
C3	0.54382 (10)	0.2500	0.5832 (3)	0.0382 (4)	
H3	0.5635	0.2500	0.6939	0.046*	
C4	0.47569 (10)	0.2500	0.5712 (2)	0.0347 (4)	
C5	0.43407 (11)	0.2500	0.7208 (3)	0.0428 (5)	
H5	0.4517	0.2500	0.8341	0.051*	
C6	0.36779 (11)	0.2500	0.6971 (3)	0.0461 (5)	
H6	0.3408	0.2500	0.7960	0.055*	
C7	0.33908 (10)	0.2500	0.5279 (3)	0.0411 (5)	
C8	0.37829 (9)	0.2500	0.3777 (3)	0.0361 (4)	
C9	0.44701 (9)	0.2500	0.3993 (2)	0.0322 (4)	
C10	0.65428 (11)	0.2500	0.4503 (3)	0.0531 (6)	
H10	0.6787	0.2500	0.3467	0.064*	
C11	0.26571 (11)	0.2500	0.5126 (4)	0.0583 (6)	
H11A	0.2468	0.2766	0.6262	0.087*	0.50
H11B	0.2512	0.1227	0.4713	0.087*	0.50
H11C	0.2524	0.3507	0.4305	0.087*	0.50
C12	0.34939 (11)	0.2500	0.1950 (3)	0.0532 (6)	
H12A	0.3794	0.1882	0.1144	0.080*	0.50
H12B	0.3414	0.3842	0.1583	0.080*	0.50
H12C	0.3090	0.1776	0.1957	0.080*	0.50

### Atomic displacement parameters (Å^2^)

**Table:**

	*U*^11^	*U*^22^	*U*^33^	*U*^12^	*U*^13^	*U*^23^
Cl1	0.0424 (3)	0.0881 (5)	0.0400 (3)	0.000	0.0087 (2)	0.000
N1	0.0355 (8)	0.0437 (8)	0.0298 (7)	0.000	−0.0008 (6)	0.000
O1	0.0480 (10)	0.0820 (12)	0.0716 (12)	0.000	−0.0252 (9)	0.000
C1	0.0356 (10)	0.0415 (9)	0.0336 (9)	0.000	0.0018 (7)	0.000
C2	0.0344 (10)	0.0387 (9)	0.0423 (11)	0.000	−0.0058 (7)	0.000
C3	0.0417 (11)	0.0389 (9)	0.0341 (9)	0.000	−0.0097 (7)	0.000
C4	0.0411 (10)	0.0344 (9)	0.0286 (9)	0.000	−0.0022 (7)	0.000
C5	0.0497 (12)	0.0513 (11)	0.0275 (9)	0.000	0.0003 (8)	0.000
C6	0.0483 (12)	0.0556 (11)	0.0343 (10)	0.000	0.0078 (9)	0.000
C7	0.0357 (10)	0.0453 (10)	0.0423 (10)	0.000	0.0028 (8)	0.000
C8	0.0351 (10)	0.0386 (9)	0.0346 (9)	0.000	−0.0021 (7)	0.000
C9	0.0361 (9)	0.0324 (8)	0.0281 (8)	0.000	−0.0014 (7)	0.000
C10	0.0375 (11)	0.0623 (13)	0.0595 (14)	0.000	−0.0064 (10)	0.000
C11	0.0367 (11)	0.0783 (16)	0.0600 (15)	0.000	0.0085 (10)	0.000
C12	0.0395 (11)	0.0814 (15)	0.0387 (11)	0.000	−0.0054 (9)	0.000

### Geometric parameters (Å, °)

**Table:**

Cl1—C1	1.7545 (19)	C6—C7	1.409 (3)
N1—C1	1.290 (2)	C6—H6	0.9300
N1—C9	1.378 (2)	C7—C8	1.391 (3)
O1—C10	1.192 (3)	C7—C11	1.505 (3)
C1—C2	1.419 (3)	C8—C9	1.415 (3)
C2—C3	1.374 (3)	C8—C12	1.504 (3)
C2—C10	1.478 (3)	C10—H10	0.9300
C3—C4	1.396 (3)	C11—H11A	0.9600
C3—H3	0.9300	C11—H11B	0.9600
C4—C5	1.416 (3)	C11—H11C	0.9600
C4—C9	1.427 (2)	C12—H12A	0.9600
C5—C6	1.367 (3)	C12—H12B	0.9600
C5—H5	0.9300	C12—H12C	0.9600
			
C1—N1—C9	117.76 (16)	C7—C8—C9	118.58 (17)
N1—C1—C2	126.61 (17)	C7—C8—C12	121.64 (18)
N1—C1—Cl1	115.19 (14)	C9—C8—C12	119.79 (17)
C2—C1—Cl1	118.20 (15)	N1—C9—C8	118.25 (15)
C3—C2—C1	115.60 (18)	N1—C9—C4	120.82 (16)
C3—C2—C10	120.21 (18)	C8—C9—C4	120.92 (16)
C1—C2—C10	124.20 (19)	O1—C10—C2	124.4 (2)
C2—C3—C4	121.19 (17)	O1—C10—H10	117.8
C2—C3—H3	119.4	C2—C10—H10	117.8
C4—C3—H3	119.4	C7—C11—H11A	109.5
C3—C4—C5	123.21 (17)	C7—C11—H11B	109.5
C3—C4—C9	118.02 (16)	H11A—C11—H11B	109.5
C5—C4—C9	118.77 (18)	C7—C11—H11C	109.5
C6—C5—C4	119.43 (18)	H11A—C11—H11C	109.5
C6—C5—H5	120.3	H11B—C11—H11C	109.5
C4—C5—H5	120.3	C8—C12—H12A	109.5
C5—C6—C7	122.15 (18)	C8—C12—H12B	109.5
C5—C6—H6	118.9	H12A—C12—H12B	109.5
C7—C6—H6	118.9	C8—C12—H12C	109.5
C8—C7—C6	120.15 (19)	H12A—C12—H12C	109.5
C8—C7—C11	120.8 (2)	H12B—C12—H12C	109.5
C6—C7—C11	119.0 (2)		
			
C9—N1—C1—C2	0.0	C11—C7—C8—C9	180.0
C9—N1—C1—Cl1	180.0	C6—C7—C8—C12	180.0
N1—C1—C2—C3	0.0	C11—C7—C8—C12	0.0
Cl1—C1—C2—C3	180.0	C1—N1—C9—C8	180.0
N1—C1—C2—C10	180.0	C1—N1—C9—C4	0.0
Cl1—C1—C2—C10	0.0	C7—C8—C9—N1	180.0
C1—C2—C3—C4	0.0	C12—C8—C9—N1	0.0
C10—C2—C3—C4	180.0	C7—C8—C9—C4	0.0
C2—C3—C4—C5	180.0	C12—C8—C9—C4	180.0
C2—C3—C4—C9	0.0	C3—C4—C9—N1	0.0
C3—C4—C5—C6	180.0	C5—C4—C9—N1	180.0
C9—C4—C5—C6	0.0	C3—C4—C9—C8	180.0
C4—C5—C6—C7	0.0	C5—C4—C9—C8	0.0
C5—C6—C7—C8	0.0	C3—C2—C10—O1	0.0
C5—C6—C7—C11	180.0	C1—C2—C10—O1	180.0
C6—C7—C8—C9	0.0		
